# Mandatory universal masking is the key to stop COVID-19

**DOI:** 10.7189/jogh.10.020383

**Published:** 2020-12

**Authors:** Timothy PH Lin, David SC Hui, Jost B Jonas, Suber S Huang, Dennis SC Lam

**Affiliations:** 1 Faculty of Medicine, The Chinese University of Hong Kong, Hong Kong, China; 2 Stanley Ho Center for Emerging Infectious Diseases, Jockey Club School of Public Health and Primary Care, Faculty of Medicine, The Chinese University of Hong Kong, Hong Kong, China; 3 Department of Ophthalmology, Medical Faculty Mannheim, Heidelberg University, Heidelberg, Germany; 4 Retina Center of Ohio, Cleveland, USA; 5 Bascom Palmer Eye Institute, University of Miami, Miami, Florida, USA; 6 Department of Ophthalmology and Visual Sciences, The Chinese University of Hong Kong, Hong Kong, China; 7 C-MER (Shenzhen) Dennis Lam Eye Hospital, Shenzhen, Guangdong, China; 8 International Eye Research Institute of The Chinese University of Hong Kong (Shenzhen), Shenzhen, China; 9 C-MER Dennis Lam & Partners Eye Center, C-MER International Eye Care Group, Hong Kong, China

The severe acute respiratory syndrome coronavirus 2 (SARS-CoV-2), the culprit of the coronavirus disease 2019 (COVID-19), continues amplifying at a pandemic speed as the case and death tolls in the worst hit countries soared to new highs. While cases of COVID-19 surpassed 4.6 million in the United States (US), President Trump’s initial resistance to experts’ pleas of wearing masks in public areas inflicted fierce debates centered on universal masking. Internationally, governments have adopted different possible non-pharmaceutical means in an attempt to contain COVID-19 while waiting for vaccines and specific treatments [1]. Among all, we believe early implementation of mandatory universal masking is key to successful control of the pandemic.

The major routes of transmission of COVID-19 include droplets, airborne aerosols and fomites. Current scientific evidence has unanimously concluded that masking is an effective means of preventing SARS-CoV-2 transmission [2,3]. However, reluctance to masking remains a common phenomenon among Western countries amidst the pandemic attributable to influences from political leaders, cultural factors and knowledge gaps. As health care personnel based in the Asia-Pacific city of Hong Kong where universal masking has been a commonplace practice since the advent of COVID-19, we believe it has played a paramount role in the impressively low case records our city has maintained despite having been hit by the initial wave of the pandemic and being one of the most densely populated areas globally [4]. We advocate strongly for mandatory universal masking, an action from the level of individual citizens which could ultimately determine the forecast of COVID-19.

The notion that universal masking is unnecessary, as proposed by Klompas et al. and shared by many alike is based on the assumption that sufficient social-distancing attenuates disease transmission and the risk is minimal despite exposure to SARS-CoV-2 given such distance is maintained [5]. We agree that in perfectly controlled experimental settings where physical distances are maintained, masking may only offer small additional benefits to prevent disease transmission. Nevertheless, in reality, when people come into close proximity with one other, for instance in confined spaces like elevators or public transport inside which adequate social distancing becomes impossible, masking can provide substantial and obvious benefits in preventing disease transmission [2,6].

In addition to protection against droplet and aerosol transmission, masking may also protect against transmission by the indirect contact route. SARS-CoV-2 has been reported to survive in the environment for up to 72 hours [7]. Both health care workers and civilians can be exposed to surfaces contaminated by the virus and possibly carry viral particles to their mucous membranes, for instance, by rubbing and touching the eye, nose and mouth, which allows inoculation and infection by the SARS-CoV-2. We believe this problem can partially be alleviated by well-fitted masks properly worn, which not only form a physical barrier between hands and mucous membranes to deter their direct contact but also serve as a conspicuous reminder to refrain people from constantly touching their faces, so that transmission by such route can be reduced.

Zhang et al. further highlighted the importance of universal masking in flattening the COVID-19 curve [3]. Their data illustrated that aggressive implementation of mandatory mass masking in the initial epicentre of Wuhan by the Chinese government is the determinant in its rapid curve flattening compared to Italy and the US, the two other epicenters of outbreak, where a universal masking policy was not in place. Furthermore, the mathematical models in the study revealed that since the implementation of mandatory masking, it alone reduced the number of infections by over 78 000 in Italy from April 6 to May 9 and over 66 000 in New York City from April 17 to May 9. It is evident and beyond disputes that universal masking has a determinant role in flattening the curve and containing the outbreak. Alternative measures like social distancing, quarantine and isolation, while having their own values, as concluded by the study, are incapable of replacing the role of masks in effectively curbing COVID-19 contrary to the common belief.

Masking is usually contemplated as a talisman and a form of self-protection in this pandemic, which shields users from contracting COVID-19. Moreover, droplets and airborne aerosols at the scale of microns are major routes of SARS-CoV-2 transmission, which are shed from infected individuals during breathing, speaking, coughing and sneezing. Latest measurements showed that intense coughs and sneezes could propel droplets >20 feet while simultaneously generating thousands of aerosols capable of travelling even further [8]. Furthermore, increasing recent evidence indicates significant airborne transmission of SARS-CoV-2. As a result, the 6-feet social distance recommended by the Centers for Disease Control and Prevention (CDC) is indeed inadequate to eliminate transmission [9], both in indoor settings where aerosols accumulate over time and outdoor environments where airflow currents transport them over long distances. Universal masking hence offers the advantage that not only healthy individuals but asymptomatic carriers are both masked. Masks form an effective barrier to minimize virus shed from infectious carriers to achieve source control so the protection for healthy citizens are maximized. Without universal masking, asymptomatic and silent carriers who continue shedding virus would greatly undermine the efforts to contain the outbreak. Universal masking is therefore an indispensable and synergistic measure to complement existing policies to effectively curb COVID-19.

Since the advent of COVID-19, the global economy has suffered tremendously. Civilians’ daily lives have faced massive disruptions. Governments have been bombarded by the daunting mission to curb the pandemic whilst minimizing financial losses and public discontent. We acknowledge the imminent and pressing need for the society to resume normal. The balance between the pandemic control and the overall needs of the society is a daunting challenge. In consideration of the evidence discussed, we believe mandatory universal masking is a good compromise to help better control of SARS-CoV-2 transmission and enable gradual reopening of economy and restoration of social activities safely. Universal masking in public areas and confined spaces ought to become a new normal in the era of COVID-19 until safe and effective vaccines and treatments become widely available.

Kabakian-Khasholian and colleagues argued against mandatory universal masking as a public health intervention for containing the COVID-19 outbreak in their recent viewpoint [10]. The authors averred that in light of a volatile political environment with a weak and newly formed government in Lebanon, conflicting recommendations from different authorities and the seemingly lack of evidence, mandatory mass masking should not be enforced for outbreak containment. We express reservations on such views. Viruses recognize no borders and less developed regions have suffered equally, if not more, as developed countries. The presence of a weak government which may be less capable of coordinating large scale public health interventions and with less resources at disposal is an even more compelling reason in favour of universal masking, which is a simple action initiated at the level of individual citizens least dependent on the governors. Indeed, the authors acknowledged that despite the dubious governance, the country did succeed in flattening the outbreak curve by incorporating mandatory universal masking as one of the interventions. Furthermore, at this juncture, both the CDC and World Health Organization have reached unanimous decisions to recommend the use of masks in public settings to prevent COVID-19 outbreak [11,12]. While there is yet to be evidence of harm due to masking, mandatory universal masking is well justified to be in the best interest of the society during this desperate period.

**Figure:**
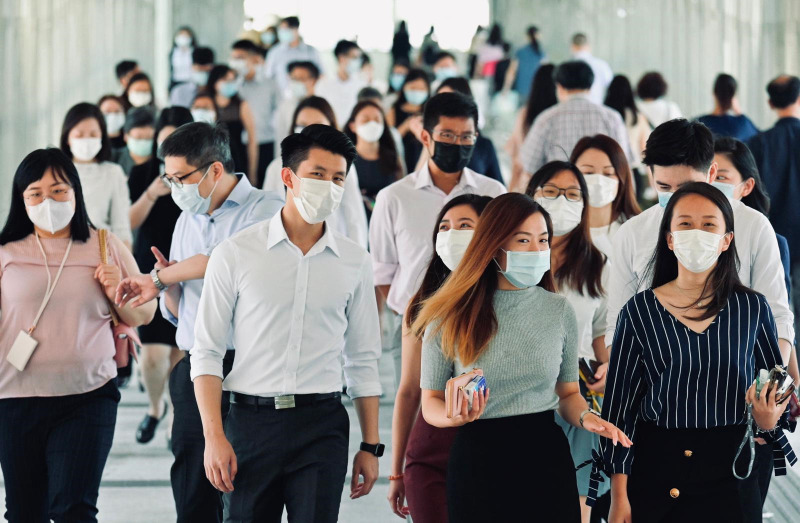
Photo: A street scene in Hong Kong during rush hours with everyone wearing masks to prevent COVID-19. (Photo courtesy of Sing Tao Daily).

We emphasize the need for masking to be mandatory. Personal freedom shall be respected in civilized societies. Nevertheless, the society overall suffers as much, if not more, from the dire consequences of this pandemic as individual citizens. In the desperate time when compliance of all people to masking is crucial to achieving outbreak containment, we believe the society’s interests must take precedence and a consensus to forgo part of our personal freedom and abide by this draconian policy is inevitable. A practical concern now lies in the potentially insufficient supply of masks when universal masking is mandatory. Reusable cloth masks recommended by the CDC is an acceptable compromise for the general public for both the purposes of source control and protection against the transmission [13]. Surgical masks and respirators with superior efficacy of aerosol filtration which offer a higher level of protection can be reserved for health care professionals while they remain in critical supplies.

It has come the epoch-making moment for political leaders and everyone to take resolute actions which are evidence-based and data-driven. Desperate moments call for desperate measures. For humanity to triumph over COVID-19, it demands our rationality to abandon the negligible personal freedom and misbeliefs for prompt and aggressive implementation of mandatory universal masking as a core intervention against one of the worst pandemics in human history.

## References

[1] Wong RLM Lai KHW Huang SS Jonas JB Lam DSC COVID-19 Pandemic: Ways Forward. Asia Pac J Ophthalmol (Phila). 2020;9:59-60. 10.1097/APO.0000000000000283 32349111 PMC7227197

[2] Chu DK Akl EA Duda S Solo K Yaacoub S Schunemann HJ Physical distancing, face masks, and eye protection to prevent person-to-person transmission of SARS-CoV-2 and COVID-19: a systematic review and meta-analysis. Lancet. 2020;395:1973-87. 10.1016/S0140-6736(20)31142-9 32497510 PMC7263814

[3] Zhang R Li Y Zhang AL Wang Y Molina MJ Identifying airborne transmission as the dominant route for the spread of COVID-19. Proc Natl Acad Sci U S A. 2020;117:14857-63. 10.1073/pnas.2009637117 32527856 PMC7334447

[4] Wan KH Lin TPH Ko CN Lam DSC Impact of COVID-19 on Ophthalmology and Future Practice of Medicine. Asia Pac J Ophthalmol (Phila). 2020;9:279-80. 32739939 10.1097/APO.0000000000000305

[5] Klompas M Morris CA Sinclair J Pearson M Shenoy ES Universal Masking in Hospitals in the Covid-19 Era. N Engl J Med. 2020;382:e63. 10.1056/NEJMp2006372 32237672

[6] Leung NHL Chu DKW Shiu EYC Chan KH McDevitt JJ Hau BJP Respiratory virus shedding in exhaled breath and efficacy of face masks. Nat Med. 2020;26:676-80. 10.1038/s41591-020-0843-2 32371934 PMC8238571

[7] van Doremalen N Bushmaker T Morris DH Holbrook MG Gamble A Williamson BN Aerosol and Surface Stability of SARS-CoV-2 as Compared with SARS-CoV-1. N Engl J Med. 2020;382:1564-7. 10.1056/NEJMc2004973 32182409 PMC7121658

[8] Morawska L Cao J Airborne transmission of SARS-CoV-2: The world should face the reality. Environ Int. 2020;139:105730. 10.1016/j.envint.2020.105730 32294574 PMC7151430

[9] Centers for Disease Control and Prevention. Social Distancing. 2020. Available: https://www.cdc.gov/coronavirus/2019-ncov/prevent-getting-sick/social-distancing.html. Accessed: 1 August 2020.

[10] Kabakian-Khasholian T Makhoul J Bardus M To wear or not to wear a mask in the COVID-19 era? The broken bridge between recommendations and implementation in Lebanon. J Glob Health. 2020;2:020311 10.7189/jogh.10.020311 PMC7535134 33110515

[11] Centers for Disease Control and Prevention. Considerations for Wearing Masks. 2020. Available: https://www.cdc.gov/coronavirus/2019-ncov/prevent-getting-sick/cloth-face-cover-guidance.html. Accessed: 1 August 2020.

[12] World Health Organization. Advice on the use of masks in the context of COVID-19: interim guidance, 5 June 2020. 2020. Available: https://apps.who.int/iris/handle/10665/332293?search-result=true&query=Advice+on+the+use+of+masks+in+the+context+of+COVID-19&scope=&rpp=10&sort_by=score&order=desc. Accessed: 1 August 2020.

[13] Centers for Disease Control and Prevention. How to make masks. 2020. Available: https://www.cdc.gov/coronavirus/2019-ncov/prevent-getting-sick/how-to-make-cloth-face-covering.html. Accessed: 1 August 2020.

